# Work-Related Stress, Burnout and Disordered Eating in Healthcare Professionals: A Cross-Sectional Study

**DOI:** 10.1192/j.eurpsy.2026.11372

**Published:** 2026-07-31

**Authors:** F. Pastore, E. Albino, E. Domenicone, L. Domeneck, S. Domeneck Nissan, A. Litta

**Affiliations:** 1 Department of Translational Biomedicine and Neuroscience (DiBraiN), University of Bari “Aldo Moro”; 2 Department of Precision and Regenerative Medicine and Ionian Area (DiMePRe-J), University of Bari “Aldo Moro”, Bari; 3 Department of Medical Specialties – Neurology Unit, San Filippo Neri Hospital, ASL Roma 1, Roma; 4 Interdisciplinary Department of Medicine, University of Bari “Aldo Moro”, Bari; 5 Mental Health Department, Asl Taranto, Taranto, Italy

## Abstract

**Introduction:**

Binge Eating Disorder (BED) is among the most common eating disorders and has been linked to stress, emotional dysregulation and maladaptive coping. Healthcare professionals, often exposed to demanding work environments and chronic stressors, may be especially vulnerable to developing dysfunctional eating behaviours. Despite this, the relationship between occupational stress, burnout and binge eating symptoms in this population remains insufficiently investigated.

**Objectives:**

This study set out to explore how work-related stress and emotional exhaustion may be connected to binge eating tendencies among healthcare professionals in Italy, with the aim of better understanding risk patterns and informing possible preventive strategies.

**Methods:**

A cross-sectional online survey was conducted between May and July 2024 with 312 healthcare professionals. Participants completed a structured questionnaire including sociodemographic data, stress-related variables, items from the Emotional Exhaustion subscale of the Maslach Burnout Inventory (MBI) and the Binge Eating Scale (BES). Data were analysed using non-parametric tests and correlation analyses.

**Results:**

The sample was predominantly female (81.7%), with a mean age of 37.6 years. Nurses (31.9%), physicians (19.0%) and professional educators (12.3%) were the most represented categories, followed by smaller groups of psychologists, auxiliary staff and social workers. A history of eating disorders was reported by 20% of participants, while 60% reported current stress or anxiety. Higher BES scores were significantly associated with stress-related variables, including anxiety, emotional exhaustion, eating during work breaks and vending machine use (p < 0.005). Strong correlations were observed between BES scores and burnout symptoms such as fatigue, emotional drain and impaired coping. Participants frequently perceived stress as directly influencing their eating behaviours.

**Conclusions:**

Healthcare professionals exposed to high work-related stress and burnout symptoms appear at increased risk for binge eating tendencies. Preventive interventions—such as institutional stress management programs, psychological support and improved access to healthy food at the workplace—may mitigate maladaptive eating behaviours and support well-being in this vulnerable population.

**Disclosure of Interest:**

None Declared

